# Cross‐Talk Between Histamine H_2_ Receptor and Glucocorticoid Receptor: Potential Implications in Acute Myeloid Leukemia Treatment

**DOI:** 10.1002/prp2.70314

**Published:** 2026-09-14

**Authors:** Valeria Torralba‐Agu, Natalia Fernández, Carina Shayo, Carlos Davio, C. Daniel Zappia, Federico Monczor

**Affiliations:** ^1^ Consejo Nacional de Investigaciones Científicas y Técnicas (CONICET), Instituto de Investigaciones Farmacológicas (ININFA) Universidad de Buenos Aires Buenos Aires Argentina; ^2^ Facultad de Farmacia y Bioquímica Universidad de Buenos Aires Buenos Aires Argentina; ^3^ Laboratorio de Patología y Farmacología Molecular, Instituto de Biología y Medicina Experimental CONICET Buenos Aires Argentina

**Keywords:** cell signaling, glucocorticoids, histamine, leukemic cell proliferation, leukemic cell resistance, receptor crosstalk

## Abstract

This study investigates the impact of histamine H_2_ receptor activation on glucocorticoid receptor (GR)‐mediated transcriptional activity, as well as its effects on leukemic cell proliferation and chemotherapy resistance. We demonstrate that activation of the histamine H_2_ receptor by its specific agonist, amthamine, enhances GR transcriptional activity induced by dexamethasone in both artificial promoter‐driven luciferase reporter assays and endogenous GR‐responsive genes. Mechanistically, histamine H_2_ receptor signaling exerts a dual regulatory effect: an inhibitory influence mediated by cAMP and a stimulatory effect through inhibition of the PI3K‐AKT–mTOR‐S6K pathway and activation of the ERK pathway. Furthermore, while dexamethasone reduces leukemic cell proliferation at high doses, it increases it at lower concentrations, an effect that is abrogated by amthamine. Moreover, the combination of the glucocorticoid and the H_2_ agonist reestablishes leukemic cell sensitivity to cytarabine, thereby restoring its cytotoxicity. Together, these findings offer insight into the molecular mechanisms and therapeutic potential of the interplay between histamine H_2_ ligands and GR agonists, and may help guide dosage optimization and mitigate the well‐known side effects of glucocorticoid therapy, with potential clinical implications for the treatment of acute myeloid leukemia.

**Abbreviations**
ALL: acute lymphoblastic leukemia
AML: acute myeloid leukemia
ANXA1: Annexin A1
GILZ: Glucocorticoid induced leucine zipper
GR: glucocorticoid receptor
H_1_R: histamine H_1_ receptor
H_2_R: histamine H_2_ receptor
HDC: Histamine dihydrochloride
MKP1: Mitogen‐activated protein Kinase Phosphatase 1

## 1 Introduction

The glucocorticoid receptor (GR) is a nuclear receptor that, following ligand binding, translocates from the cytoplasm to the nucleus and modulates gene expression either directly by binding to specific DNA regions, or indirectly, through interaction with other transcription factors [1, 2]. In this way, glucocorticoids regulate nearly 20% of genome activity [3].

Synthetic glucocorticoids, widely prescribed for their anti‐inflammatory and immunomodulatory properties, are mainly used to treat allergic, autoimmune, and chronic inflammatory diseases. In oncology, their use as antileukemic agents is well established but has historically been confined to malignancies of lymphoid origin, such as acute lymphoblastic leukemia (ALL), lymphomas, and multiple myeloma, where their ability to trigger apoptosis in lymphoid cells makes them a therapeutic backbone [4]. In contrast, acute myeloid leukemia (AML), a heterogeneous hematologic malignancy characterized by the clonal proliferation and accumulation of immature myeloid blasts in the bone marrow [5], has classically been regarded as glucocorticoid‐resistant, and glucocorticoids are not included in current clinical practice guidelines for its treatment [6]. AML management continues to rely primarily on poorly tolerated chemotherapy with cytarabine and anthracyclines [7], and apart from the supportive control of differentiation syndrome, glucocorticoids have no established role in its antileukemic regimen. However, several reports have shown that glucocorticoids such as dexamethasone and prednisolone can promote differentiation and sensitize AML cells to chemotherapeutic agents [8, 9, 10, 11, 12].

Nonetheless, the extensive use of glucocorticoids is constrained by adverse effects, prompting efforts to refine GR activity modulation toward more targeted treatments with fewer side effects [13, 14]. GR activity is influenced by multiple signaling pathways. Crosstalk has been reported between this receptor and nuclear receptors (e.g., PPARγ, LXR, and RAR) [15] as well as GPCRs (e.g., β2‐adrenergic, somatostatin, and melatonin receptors) [16, 17, 18]. Our laboratory previously demonstrated that histamine and antihistamines acting on the histamine H_1_ receptor (H_1_R) enhance glucocorticoid receptor transcriptional activity. This regulation is intrinsically dual, involving potentiation via G‐protein βγ subunits and inhibition through the Gαq‐PLC pathway [19]. Additionally, we showed that antihistamines amplify glucocorticoids' anti‐inflammatory effects in a ligand‐, cell‐, and gene‐dependent manner [20].

Given that histamine can modulate GR activity through H_1_R, in the present work we investigated the role of H_2_R signaling on GR signaling. This may provide insights into potential synergistic or modulatory mechanisms relevant to AML treatment, with the aim of identifying new therapeutic strategies capable of improving clinical outcomes while reducing treatment‐related toxicity [5, 6].

Histamine dihydrochloride (HDC) has been approved in combination with interleukin‐2 (IL‐2) as maintenance therapy to prevent relapse in AML [21, 22, 23]. This effect has been shown to be mediated through H_2_R activation, which inhibits NADPH oxidase–dependent ROS production in myeloid cells and increases intracellular cAMP. Consequently, histamine enhances the survival of lymphocytes and natural killer (NK) cells with antineoplastic activity. In parallel, H_2_R activation has been shown to promote AML cell differentiation, offering an additional therapeutic benefit beyond its immunomodulatory effects [21, 24]. Together, these effects suggest that histamine signaling through H_2_R could contribute to restoring normal hematopoietic differentiation and improving immune‐mediated antileukemic responses [22].

Based on these observations, in this study, we evaluated how H_2_R activation impacts glucocorticoid receptor‐mediated transcriptional activity. We observed that the H_2_R agonist amthamine enhances glucocorticoid receptor‐driven transcriptional activity induced by dexamethasone on both artificial promoters and endogenous genes. We also examined the effects of co‐treatment on leukemic cell proliferation and in the context of cytarabine exposure, in both sensitive and resistant cells, finding that co‐treatment with both ligands re‐sensitized the latter. These findings suggest that H_2_R agonists may be worth exploring as adjuvants in combination therapies with glucocorticoids for AML, warranting further validation. This combination may enable the use of lower glucocorticoid doses, reducing adverse effects while maintaining or enhancing antileukemic efficacy, as well as resensitizing cytarabine‐resistant cells.

## 2 Materials and Methods

### 2.1 Materials

DMEM and RPMI media, antibiotics, phosphate‐buffered saline (PBS), bovine serum albumin (BSA), histamine dihydrochloride, amthamine dihydrobromide, dexamethasone, gallein, forskolin, wortmannin, UO126 and H89 were obtained from Sigma Chemical Company (St. Louis, MO). Amthamine dihydrobromide and PD 98059 were acquired from Tocris Cookson Inc. (Ballwin, MO). Rapamycin was obtained from LC Laboratories (Woburn, MA). Fetal bovine serum (FBS) was purchased from Natocor (Córdoba, Argentina). Cytosine β‐D‐arabinofuranoside (cytarabine, Ara‐C) was purchased from Microsules Bernabó (Buenos Aires, Argentina). All other chemicals were of analytical grade and obtained from standard sources.

### 2.2 Plasmid Constructions

pRSV‐GR was cloned by Dr. Keith Yamamoto. It includes a full length GR cDNA which was subcloned downstream to the promiscous Rous Scarcoma Virus (RSV) promoter [25]. Human histamine H_2_ receptor cDNA was cloned into the *Spe*I and *Eco*RV sites of the eukaryotic expression vector pCEFL as described in [26]. TAT3 contains 3xGRE derived from tyrosine aminotransferase gene. The core palindrome sequence for a single GRE is 5′‐AGAACAnnnTGTTCT‐3′ [19]. pCMV‐Myc‐N‐EPAC was cloned from pMT2SMHA‐Epac using a SalI‐BglII fragment, which contains the DEP, CBD, and REM domains (1–1270 nucleotides) of EPAC and pMT2‐HA‐Rap1GAP was generated by subcloning full‐length RapGAP as a SalI/BglII fragment into pMT2HA plasmids and was provided by Dr. Omar Coso (Department of Physiology and Molecular Biology, Universidad de Buenos Aires, Argentina) [27].

### 2.3 Cell Culture

HEK293T (human embryonic kidney) cells (ATCC CRL‐3216; RRID: CVCL_0063) were cultured in Dulbecco's modified Eagle's medium (DMEM) [28]. U937 (ATCC CRL‐1593.2; RRID: CVCL_0007) and U937‐H2R (human leukemia cell lines) were cultured in RPMI 1640 medium. The U937‐H2R clone was previously generated in our laboratory [26]. All cells used were contamination‐free. All media were supplemented with 10% fetal calf serum and 5 μg/mL gentamicin, and cells were incubated at 37°C in a humidified atmosphere containing 5% CO_2_. Cytarabine‐resistant U937 cells were generated following the protocol described by Malani et al. [29], with minor modifications. Briefly, U937 cells were exposed to an initial cytarabine (Ara‐C) concentration of 20 nM. The drug concentration was subsequently doubled each time the treated cells exhibited a proliferation rate comparable to that of the untreated parental culture, until a final concentration of 640 nM was achieved. This clone was named U937‐640R.

### 2.4 Cell Transfection and Gene Reporter Assay

HEK293T seeded on 24‐well plates (2.5 × 10^5^ cells/600 μL) were co‐transfected using the K2 Transfection System (Biontex, Munich, Germany) with the TAT3‐Luc luciferase reporter plasmid, pCEFL‐H2R and pRSV‐GR according to the manufacturer's instructions. In some experiments, cells were also co‐transfected with the plasmid constructs indicated in the corresponding figure or an empty vector to maintain an equal amount of total DNA. After 4 h, cells were seeded in 96‐well plates, and 24 h later cells were starved overnight and then stimulated with diverse agents. After a kinetic assessment, luciferase activity was measured at the optimal time of 24 h later using the Steady‐Glo Luciferase Assay System according to the manufacturer's instructions (Promega Biosciences Inc. San Luis Obispo, CA, USA) using a FlexStation 3 Multi‐Mode Microplate Reader (Molecular Devices LLC). Experimental reporter activity was normalized to control activity.

### 2.5 Quantitative Real‐Time PCR

Total RNA was isolated from 2 × 10^6^ U937 cells using Quick‐Zol reagent (Kalium Technologies) following the manufacturer's instructions. To eliminate potential DNA contamination from the samples prior to quantification by PCR, samples were subjected to DNase treatment. Each RNA sample received 2 μL of Baseline‐Zero solution and 1 μL of DNase, and the resulting mixture was incubated for 20 min at 37°C. Subsequently, 2 μL of Stop Solution were added, and samples were incubated for 10 min at 65°C before being stored at −80°C until further use. 1 μg of total RNA was reverse‐transcribed using the High‐Capacity cDNA Reverse Transcription kit (Applied Biosystems, Beverly, MA, USA) with random primers for the first‐strand cDNA synthesis. Quantitative real‐time PCR (qPCR) was performed in triplicate on the 7500 Fast Real‐time PCR System (Bio‐Rad, Hercules, CA, USA) using the resulting cDNA, the HOT FIREPol EvaGreen qPCR Mix Plus (Solis BioDyne, Tartu, Estonia) for product detection, and the following primers: human GILZ (glucocorticoid induced leucine zipper; NM_001015881.1) forward, 5′‐AATGCGGCCACGGATG‐3′ and reverse, 5′‐GGACTTCACGTTTCAGTGGACA‐3′; human MKP‐1 (mitogen‐activated protein kinase phosphatase 1; NM_004417.4); human ANXA1 (Annexin‐A1; NM_000700.2) forward, 5′‐CATGTGAATGCCATCCAGGA‐3′ and reverse 5′‐GGAGGTCAAACATTTCTGAGATGA‐3′; human CD14 (Monocyte differentiation antigen; NM_000591) forward, 5′‐CTGGAACAGGTGCCTAAAGGAC‐3′ and reverse 5′‐GTCCAGTGTCAGGTTATCCACC‐3′; human GADD45 β (Growth Arrest and DNA Damage Inducible beta; NM_015675.4) forward, 5′‐GCTCTCTGGCTCGGATTTTGC‐3′ and reverse 5′‐GGTCACCGTCTGCATCTTCTG‐3′; and human CDKN1A (Cyclin Dependent Kinase Inhibitor 1A; NM_000389) forward, 5′‐TGTACCCTTGTGCCTCGCTC‐3′ and reverse 5′‐GAAGATCAGCCGGCGTTTGG‐3′; human β‐Actin (βAct) forward, 5′‐GGACTTCGAGCAAGAGATGG‐3′ and reverse 5′‐AGCACTGTGTTGGCGTACAG‐3′. The cDNA was amplified by 45 cycles of denaturing (30 s at 95°C), annealing (30 s at 60°C), and extension (30 s at 72°C) steps. The specificity of each primer set was monitored by analyzing the dissociation curve, and the relative gene quantification was performed using the comparative ΔΔCt method using β‐Actin as the housekeeping gene. The suitability of the reference gene was confirmed using geNorm [30] and BestKeeper [31] algorithms.

### 2.6 Western Blot

Cells (4.5 × 10^5^ cells/2 mL, seeded in a 6‐well plate) were lysed in 50 mM Tris–HCl, pH 6.8, 2% SDS, 100 mM 2‐mercaptoethanol, 10% glycerol, and 0.05% bromophenol blue and sonicated to shear DNA. Total cell lysates were resolved by 10% SDS‐PAGE, blotted, and incubated with primary antibodies anti‐ERK1/2 (rabbit polyclonal; cat #SC‐94; Lot#I2512; wc: 1:1000), ‐pERK1/2 (mouse IgG_2a_; cat #SC‐7383; Lot#H2410; wc: 1:500), ‐Akt1/2/3 (mouse IgG; cat. #SC‐81434; Lot#G1421; wc: 1:200), ‐pAkt1/2/3 (rabbit IgG; cat. #SC‐7985; Lot#I0211; wc: 1:1000), ‐S6K (mouse IgG_1_; cat #SC‐8418; Lot#E3117; wc: 1:1000), and ‐pS6K (mouse IgG_1_; Ser434; cat #SC‐8416; Lot#G1917; wc: 1:500), from Santa Cruz Biotechnology, CA, USA. After incubation for 1 h at room temperature with a horseradish peroxidase‐conjugated anti‐rabbit antibody or anti‐mouse antibody (Vector Laboratories, CA), reaction products were revealed by enhanced chemiluminescence (ECL Plus, Pierce, Rockford, IL, USA) following the manufacturer's instructions (Amersham Life Science, England). Results were visualized by autoradiography or with the Sapphire AZURE Biosystems Imaging Systems equipment and quantified using ImageJ software (NIH, Bethesda, Maryland, USA).

### 2.7 cAMP Measurement

For determination of intracellular cAMP levels, 2 × 10^5^ cells were washed and resuspended in fresh medium containing 1 mM IBMX, incubated for 3 min, and then exposed to increasing concentrations of amthamine for 10 min. The reaction was stopped by ethanol addition followed by centrifugation at 2000 × ***g*** for 5 min. The ethanol phase was then dried, and the residue resuspended in 50 mM Tris–HCl pH 7.4, 0.1% BSA. cAMP content was determined by competition of [^3^H] cAMP for PKA [26].

### 2.8 Cell Growth Experiments

U937, U937‐H2R and cytarabine‐resistant U937 cells were seeded (1 × 10^5^ cells/600 μL) in 24‐well plates in exponential growth phase and treated with the different reagents at the times and concentrations indicated. After 96 h of treatment, cell number was determined by counting in a Neubauer chamber under a bright‐field microscope and normalized first to day zero of each well and then to the control group. Experiments were performed in triplicate in at least three independent assays.

### 2.9 Data Analysis

Curve‐fitting and statistical analysis were performed using GraphPad Prism 8.0 (GraphPAD Software for Science, San Diego, CA, USA). Cell proliferation dose–response curves were constrained at their upper and lower plateaus to facilitate graphical comparison of EC50 values. Results are expressed as mean ± SEM. Parametric statistical analysis was performed using one‐ or two‐way ANOVA, followed by Bonferroni post hoc multiple comparisons. Differences were considered significant at *p* < 0.05. Experiments were conducted in triplicate in at least three independent trials.

## 3 Results

### 3.1 Amthamine Potentiates Dexamethasone‐Induced Glucocorticoid Receptor Transcriptional Activity Through a Composite Mechanism

**FIGURE 1:**
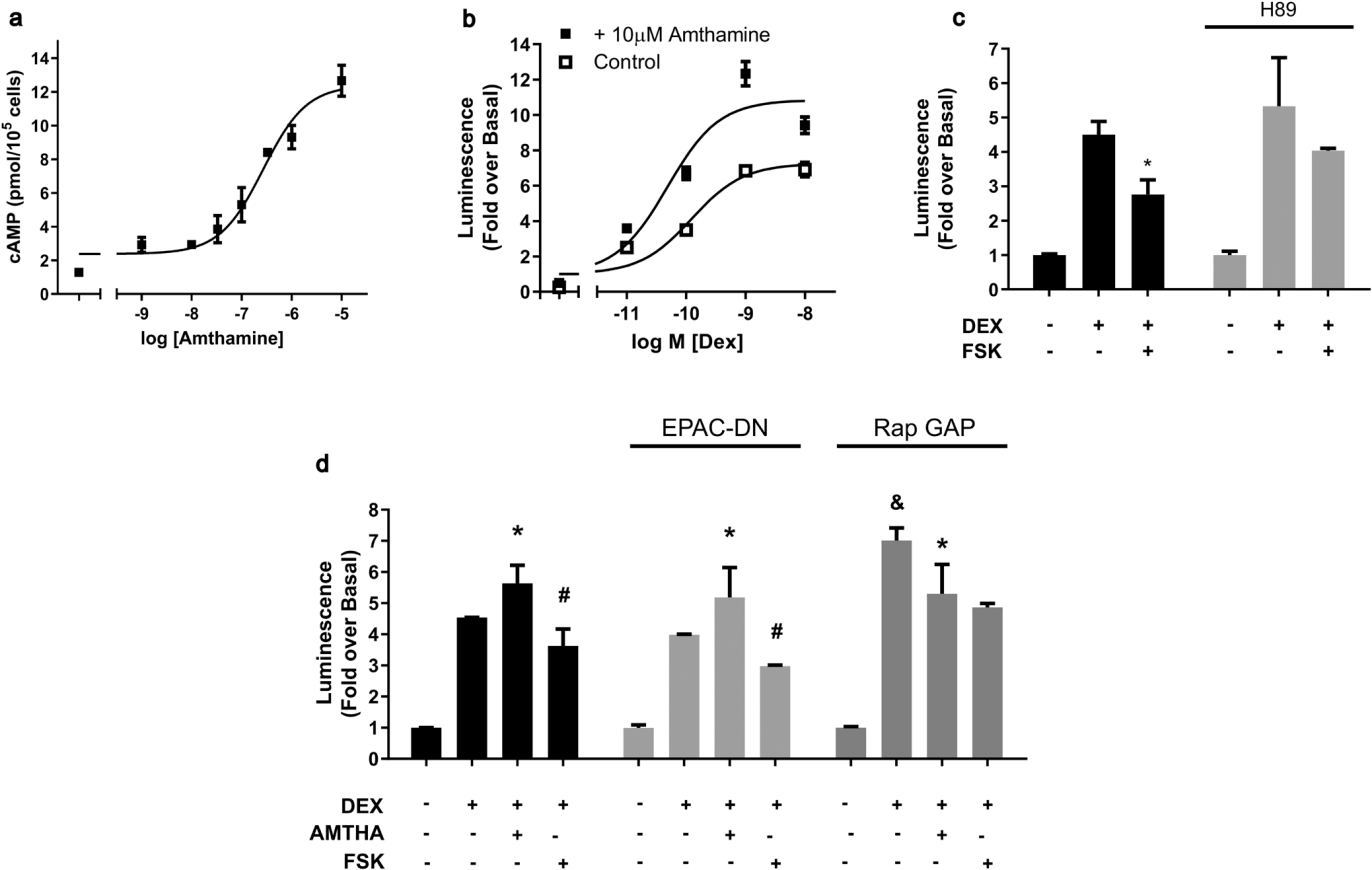
Histamine H_2_ receptor agonist activation and its effect on glucocorticoid receptor transcriptional activity. HEK‐293T cells co‐transfected with the reporter TAT3‐Luc, histamine H_2_ receptor, and glucocorticoid receptor coding plasmids (a) were exposed for 10 min to increasing concentrations of Amthamine at 37°C in the presence of 1 mM IBMX. cAMP levels were determined as detailed in the “Materials and Methods” section. Data represent the mean ± SEM of assay duplicates. Similar results were obtained in six independent experiments. (b) Cells were treated for 10 min with 10 μM amthamine (filled squares), or not (empty squares), and incubated with increasing concentrations of dexamethasone (DEX). Luciferase activity was determined as described in the methods section. Data represent the mean ± SEM of assay triplicates. Similar results were obtained in three independent experiments. (c) Cells were pre‐treated (gray bars), or not (black bars), with 1 μM H89 and then incubated with 25 μM forskolin and dexamethasone as indicated. Luciferase activity was determined as described in the methods section. Results are mean ± SEM of three independent experiments performed in triplicates. (d) Cells were cotransfected with an EPAC dominant negative construct or Rap‐GAP coding plasmids, were preincubated with 25 μM forskolin or 10 μM amthamine for 10 min and then incubated with dexamethasone. Luciferase activity was determined as described in the methods section. Results are mean ± SEM of three independent experiments performed in triplicates. *p < 0.05 respect to DEX; #*p* < 0.05 respect to DEX + AMTHA; and &*p* < 0.05 respect to control DEX.

In order to evaluate the effect of histamine H_2_ receptor signaling on glucocorticoid receptor transcriptional activity, we co‐transfected HEK293T cells with plasmids coding for the GR, the H_2_R and a TAT3 promoter‐luciferase reporter system. Activation of H_2_R leads to dissociation of the Gαs subunit from the Gβγ dimer, with canonical Gαs‐mediated activation of adenylyl cyclase increasing cAMP levels [32]. Consistently, amthamine induced a dose‐dependent increase in cAMP levels (Figure 1a). Cells were stimulated with different concentrations of dexamethasone for 24 h, with or without 10 μM amthamine pre‐treatment for 10 min. Dexamethasone alone induced a concentration‐dependent increase in luciferase activity, and amthamine pre‐treatment increased its maximal effect 1.5‐fold (7.25 ± 0.41 vs. 10.85 ± 0.85) without significantly affecting its pEC_50_ (9.9 ± 0.16 vs. 10.31 ± 0.25; Figure 1b).

We next investigated which H_2_R‐modulated signaling pathways underlie this effect of amthamine. Although amthamine treatment produced a concentration‐dependent increase in intracellular cAMP levels, this increase was not responsible for the potentiation of GR response, as it could not be mimicked by a direct activation of adenylyl cyclase (Figure 1c). On the contrary, pre‐incubation with 25 μM forskolin reduced dexamethasone‐induced GR activation, suggesting that the cAMP pathway inhibits GR activity. This inhibitory effect was reversed by H89, a protein kinase A (PKA) inhibitor, indicating that PKA is the primary mediator of cAMP's negative action on GR transcriptional activity (Figure 1c).

We then explored the roles of two other cAMP effectors: EPAC and Rap1. The dominant‐negative mutant of EPAC (EPAC‐DN) caused no change in the effects of amthamine and forskolin on dexamethasone‐induced activity, ruling out EPAC's involvement. Conversely, transfection with a Rap GAP, which inactivates Rap1, increased dexamethasone‐induced GR activity. Forskolin's effect was unchanged, indicating that Rap1 is not involved in the cAMP inhibitory effect. However, amthamine's effect switched from stimulatory to inhibitory, suggesting that Rap1 participates in amthamine‐mediated potentiation of GR activity rather than in the negative effect of cAMP (Figure 1d).

**FIGURE 2:**
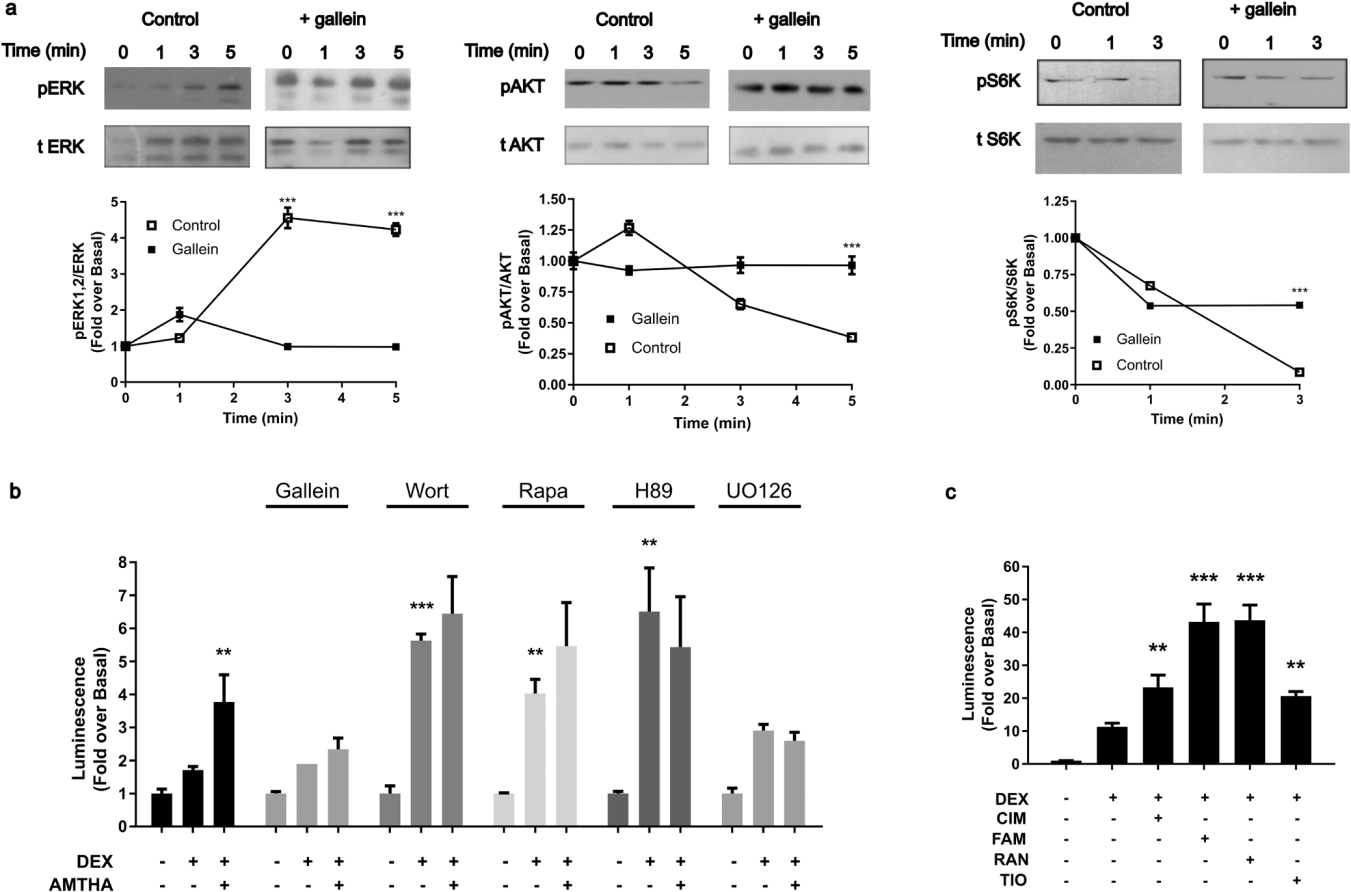
Effect of histamine H_2_ receptor triggered pathways on Glucocorticoid receptor activity potentiation. (a) HEK‐293T cells co‐transfected with the reporter TAT3‐Luc, histamine H_2_ receptor, and glucocorticoid receptor coding plasmids were treated (filled squares) or not (empty squares) with 10 μM gallein and subjected to 10 μM amthamine treatment for 1, 3 or 5 min. Whole‐cell lysates were subjected to 12% SDS polyacrylamide gel electrophoresis, followed by Western blotting with polyclonal purified sera against pERK, pAKT, or pS6K; or sera against total proteins as indicated in the methodology section. A cropped section of the membrane ranging from 30 to 60 kDa of a representative experiment is shown (right panel). Densitometric analysis was performed with ImageJ as described in the methods section (left panel). Results are mean ± SEM of three independent experiments performed. Biological replicates are depicted as Supporting Information. (b) Cells were treated with 10 μM gallein, 1 μM wortmannin, 1 μM rapamycin, 1 μM H89, or 20 μM UO126 30 min before indicated treatments. Luciferase activity was determined as described in the methods section. Results are mean ± SEM of three independent experiments performed in triplicates. **p < 0.01 and ***p < 0.001 respect to control DEX. (c) Cells were treated for 10 min with 10 μM cimetidine, famotidine, ranitidine, tiotidine, or not, and incubated with 1 nM dexamethasone. Luciferase activity was determined as described in the methods section. Data represent the mean ± SEM of assay triplicates. Similar results were obtained in three independent experiments.

To further investigate how H_2_R potentiates GR activity, we evaluated the role of signaling pathways modulated by H2R via Gβγ. As previously described [33], amthamine treatment increases ERK phosphorylation and decreases PI3K and mTOR activity, consequently reducing phosphorylation of their respective substrates AKT and S6K (Figure 2a). As expected, pre‐treatment with 10 μM gallein, a Gβγ subunit signaling inhibitor, dampened amthamine‐induced modulation of the ERK, PI3K, and mTOR pathways (Figure 2a). Notably, gallein treatment also prevented amthamine potentiation of GR response to dexamethasone, indicating that this effect is downstream of the Gβγ dimer (Figure 2b). Full length blots of biological replicates are shown as Figure S1.

We next used specific pathway inhibitors to identify the role of these effectors in the amthamine‐mediated potentiation of dexamethasone‐induced GR transcriptional activity. Treatment with 20 μM UO126 (MEK inhibitor) completely abolished amthamine‐mediated enhanced GR activity. In contrast, in the presence of 1 μM wortmannin (PI3K inhibitor) or 1 μM rapamycin (mTOR inhibitor), we observed increased dexamethasone‐stimulated GR activity. This suggests that PI3K and mTOR have an inhibitory role on GR transcriptional activity, as previously described [34, 35, 36, 37]. Accordingly, the amthamine‐induced reduction of this signaling pathway explains the increase in GR transcriptional activity observed in response to dexamethasone.

These observations indicate that the H2R signaling through Gαs and by Gβγ has opposite effects. Canonical Gαs‐AC‐cAMP‐PKA activation decreases GR response to dexamethasone. In contrast, Gβγ‐mediated MEK–ERK activation and PI3K‐Akt–mTOR inactivation converge to increase the transcriptional response to dexamethasone, with the latter effect predominating. To confirm this, we evaluated the effect of clinically used H_2_R inverse agonists cimetidine, famotidine, ranitidine, as well as tiotidine, a widely used research compound, all described as ERK‐biased ligands. As expected, all four inverse agonists enhanced the effect of dexamethasone on luciferase activity. This effect could be attributed to the decrease in cAMP and increase in pERK levels described for these ligands [38, 39, 40, 41] (Figure 2c).

### 3.2 Amthamine Differentially Modulates Glucocorticoid Receptor Responses in Leukemic Cells

**FIGURE 3:**
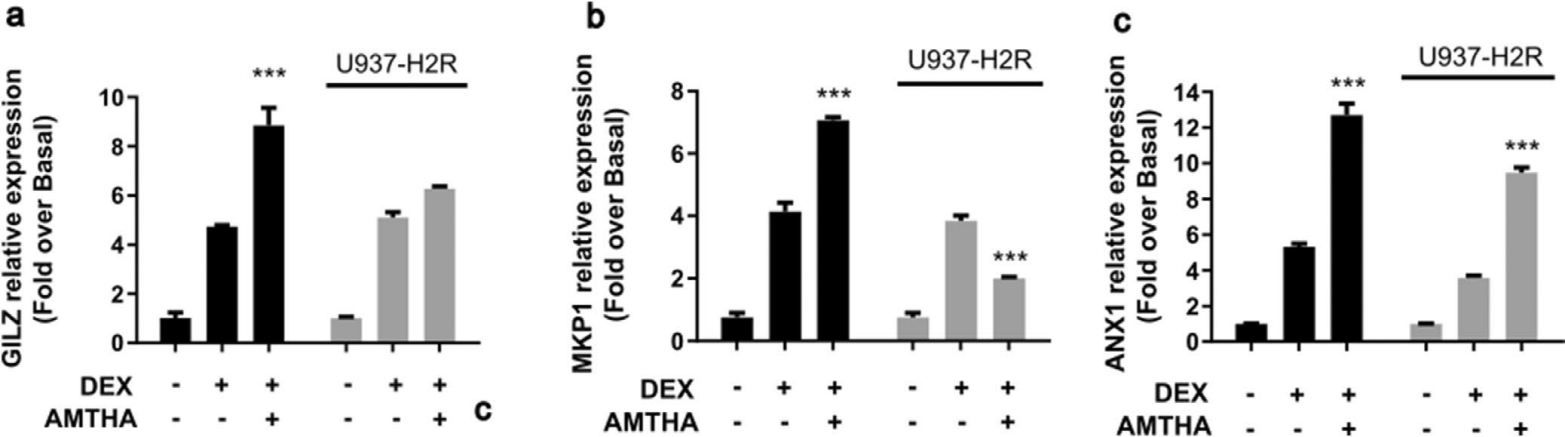
Effect of histamine H_2_ receptor agonist amthamine on dexamethasone‐induced expression of endogenous glucocorticoid receptor target genes. U937 (black bars) and U937‐H2R (gray bars) cells were incubated for 10 min with 10 μM amthamine and then treated with dexamethasone for 3 h as indicated. (a) GILZ, (b) MKP1, and (c) ANX1 mRNA levels were quantified by qPCR as described in the methods section. Results are mean+ /− SEM of at least three independent experiments performed in triplicates. ***p < 0.001 respect to DEX.

We next investigated whether H_2_R‐mediated potentiation of glucocorticoid receptor transcriptional activity on an artificial promoter could also be replicated in a pathophysiologically relevant system. To this end, we studied these effects in U937 cells, a model for acute myeloid leukemia, by measuring the expression of GILZ, MKP1, and ANXA1, three endogenous genes typically modulated by the glucocorticoid receptor. As expected, dexamethasone treatment induced all three genes in U937 cells, and co‐treatment with amthamine enhanced this effect for all the genes evaluated (Figure 3).

Notably, in a U937‐derived clone, stably overexpressing H_2_R (U937‐H_2_R) [26], although dexamethasone also induced expression of the evaluated genes, amthamine's effect persisted for ANXA1, was lost for GILZ, and switched to inhibitory for MKP1 (Figure 3). These results highlight that GR transcriptional activity is strongly dependent on both the gene and cell type studied [42] and that the effect of H_2_R stimulation on GR signaling for each gene is sensitive to the relative expression of the GPCR and the G‐protein.

**FIGURE 4:**
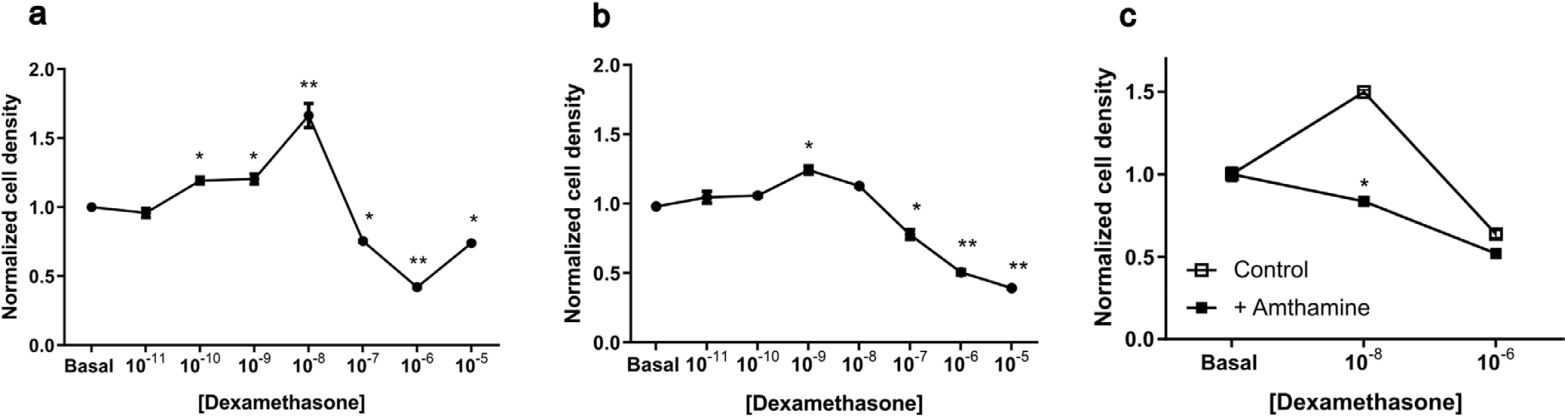
Effect of dexamethasone and amthamine on U937 cell proliferation. U937 (a) or U937‐H2R (b) cells were treated with the indicated concentrations of dexamethasone for 96 h. Cell count is presented relative to Day 0 and expressed as the mean ± SEM of three independent experiments performed in triplicates. *p < 0.05 and **p < 0.01 respect to Basal. (c) U937 cells were treated with the indicated concentrations of dexamethasone alone (empty symbols) or co‐treated with 10 μM Amthamine. Cell count is presented relative to Day 0 and expressed as the mean ± SEM of four independent experiments performed in triplicates. *p < 0.05 respect to dexamethasone alone.

Given that GR can modify leukemic cell proliferation and survival [43], we evaluated the crosstalk between H2R and GR on these cellular readouts. U937 cell proliferation was modified by dexamethasone in a concentration‐dependent manner. Low glucocorticoid concentrations (0.1–10 nM) increased cell proliferation, whereas higher concentrations (100 nM to 10 μM) diminished it. The same pattern was observed in the U937‐H_2_R clone, although the pro‐proliferative action of dexamethasone was less pronounced in this system (Figure 4a,b). Amthamine pretreatment dampened the pro‐proliferative effect of low‐dose dexamethasone but had no effect on the antiproliferative action of high doses (Figure 4c).

**FIGURE 5:**
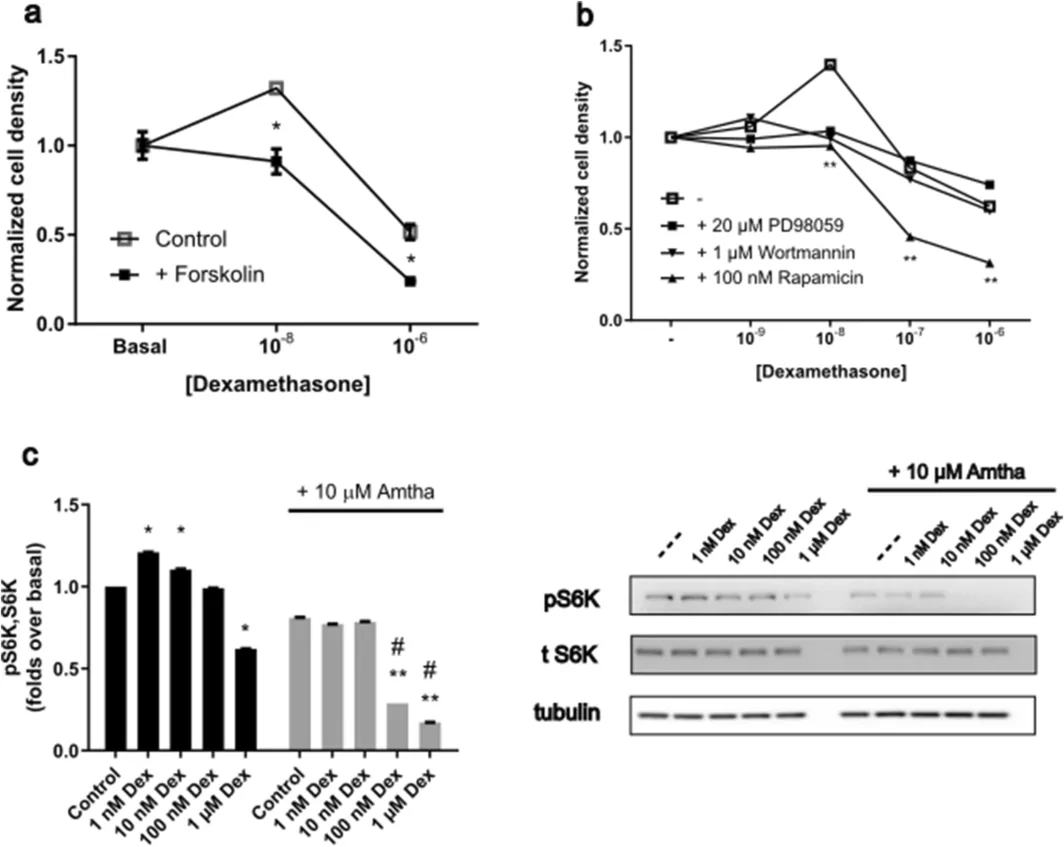
Effect of dexamethasone and kinase inhibitors on U937 cell proliferation. (a) U937 cells were treated with the indicated concentrations of dexamethasone alone (empty symbols) or co‐treated with 75 μM Forskolin. (b) U937 cells were treated with the indicated concentrations of dexamethasone alone (empty symbols) or co‐treated with 20 μM PD98059, 1 μM Wortmannin, or 100 nM Rapamicin. Cell count is presented relative to Day 0 and expressed as the mean ± SEM of four independent experiments performed in triplicates. * *p* < 0.05 and **p < 0.01 respect to dexamethasone alone. (c) Cells were treated (gray bars) or not (black bars) with 10 μM amthamine and subjected to different concentrations of dexamethasone as indicated. Whole‐cell lysates were subjected to 12% SDS polyacrylamide gel electrophoresis, followed by Western blotting with polyclonal purified sera against pS6K, tS6K or tubulin as indicated in the methodology section. A cropped section of the membrane ranging from 30 to 60 kDa of a representative experiment is shown (right panel). Densitometric analysis was performed with ImageJ as described in the methods section (left panel). Results are mean ± SEM of four independent experiments. *p < 0.05 respect to control; **p < 0.01 respect to control; # *p* < 0.05 respect to dexamethasone alone.

To investigate the basis of amthamine's modulation of dexamethasone's effect, we mimicked stimulation of the Gα‐AC‐cAMP pathway by treating the cells with forskolin and simulated Gβγ inhibition of PI3K‐Akt–mTOR signaling by incubating the cells with wortmannin or rapamycin. Remarkably, forskolin, wortmannin, and rapamycin treatment all hindered the proliferative effect of low‐dose dexamethasone, resembling amthamine effects. Notably, only rapamycin potentiates the antiproliferative effect of higher dexamethasone concentrations (Figure 5a,b).

Since both wortmannin and rapamycin precluded the proliferative effect of low concentrations of dexamethasone, we hypothesized that the PI3K‐Akt–mTOR pathway is involved in this response. Consistent with this, 10 nM dexamethasone increased p‐S6K levels, an effect blocked by 10 μM amthamine (Figure 5c; full length blots are depicted as Figure S3). These results support the conclusion that increased mTOR activity underlies the effect of glucocorticoids on cell proliferation. Furthermore, amthamine can effectively counteract this proliferative response through pharmacological modulation of this pathway.

Consistent with the effect on cell proliferation, 10 nM dexamethasone decreased expression of the cell differentiation marker CD14, while the highest concentration increases it. Similar results were obtained for the proliferation‐related genes GADD45b and CDKN1A (Figure S2).

### 3.3 Cross‐Talk Between H2R and GR Potentiates Dexamethasone Resensitization to Cytarabine in U937 Cells

**FIGURE 6:**
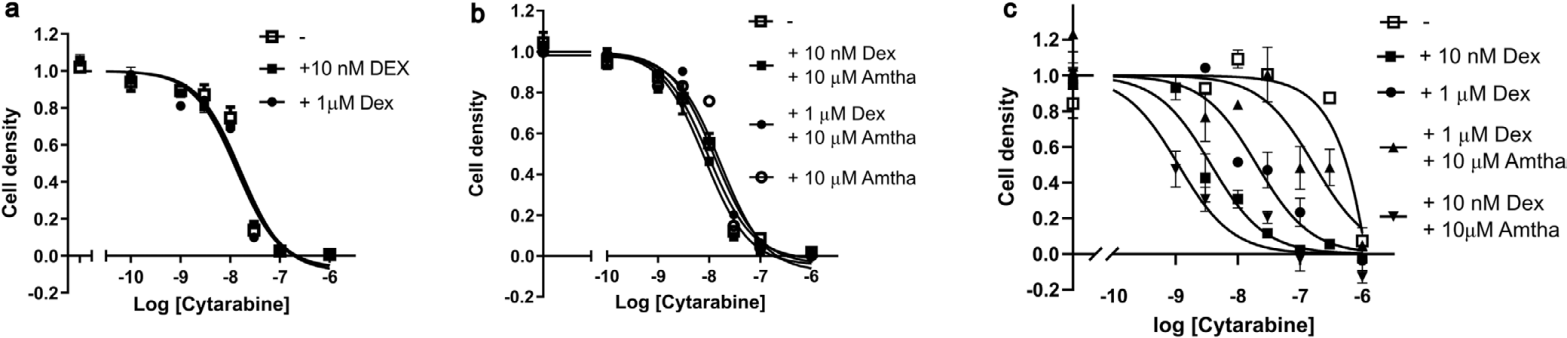
Effect of dexamethasone and amthamine on cytarabine‐induced inhibition of U937 cells. (a, b) U937 or U937‐640R (c) cells were exposed to the indicated cytarabine concentrations and pre‐exposed or not to dexamethasone and amthamine as indicated. Curve fitted parameters are shown in Table 1. Data are expressed as the mean ± SEM of three independent experiments performed in triplicates.

Considering the effect of glucocorticoids and H_2_ agonists on cell proliferation, we studied their influence on cytarabine‐induced inhibition of cell proliferation, a well‐known agent that promotes leukemic cell death. Unexpectedly, neither dexamethasone nor amthamine nor their combination significantly shifted the cytarabine concentration‐response curve (Figure 6a,b and Table 1).

Since glucocorticoids are used to reduce relapse incidence in AML, we established a cell clone resistant to cytarabine, the standard cytotoxic drug used for AML treatment. Whereas cytarabine exhibits an IC_50_ of approximately 1.5 nM in parental U937 cells, the resistant U937‐640R clone retains nearly full proliferative capacity even at 300 nM cytarabine. In this clone, both dexamethasone concentrations tested (10 nM and 1 μM) re‐sensitized the cells, reducing the cytarabine IC_50_ to 3.7 and 20 nM, respectively. In turn, 10 μM amthamine modulated dexamethasone‐induced IC_50_, producing a left‐shift of the lower dexamethasone concentration curve to 1.1 nM and a right‐shift of the higher dexamethasone concentration curve to 170 nM (Figure 6c and Table 1). These findings suggest that the combination of dexamethasone and amthamine warrants further investigation as a potential adjuvant strategy for cytarabine‐treated AM.

**TABLE 1:** Cytarabine pIC50 in U937 naïve and resistant cells. Data correspond to Figure 6 curve fitting.

	U937 cells	U937 cytarabine‐resistant cells
—	7.79 ± 0.12	N/A
+10 nM Dex	7.83 ± 0.11	8.43 ± 0.18
+1 μM Dex	7.88 ± 0.12	7.70 ± 0.18
+10 nM Dex +10 μM Amtha	8.09 ± 0.07	8.96 ± 0.16*
+1 μM Dex +10 μM Amtha	7.89 ± 0.07	6.77 ± 0.16*
+10 μM Amtha	7.78 ± 0.12	N/A

* *p* < 0.05 respect to dexamethasone only.

## 4 Discussion

Our findings show that histamine H_2_R signaling modulates GR transcriptional activity and, consequently, leukemic cell proliferation. Amthamine increased dexamethasone‐induced GR transcriptional activity through a composite mechanism involving Gβγ‐mediated potentiation and cAMP/PKA‐mediated inhibition. This mirrors our previous findings on histamine H_1_R, which similarly modulates GR activity through a dual mechanism, potentiation via Gβγ/JNK and inhibition via Gαq‐PLC [20], suggesting a common system for GR modulation by histamine receptor signaling, and adding to reports of GR crosstalk with other GPCRs [16, 17, 18]. A direct interaction between histaminergic ligands and GR is unlikely, since the effects reported here were abolished by inhibitors of signaling cascades downstream of H_2_R.

Notably, although amthamine potentiated GR transcriptional activity in both transfected and endogenous genes, when the H_2_ receptor is overexpressed, the effect of amthamine becomes gene‐specific. GR modulates gene expression through distinct mechanisms, including direct GRE‐dependent transactivation and indirect tethering to other transcription factors, each with different coactivator requirements.

We hypothesize that the divergent regulation observed in Figure 3 reflects the distinct GRE architecture of each promoter: the tandem high‐affinity GREs of GILZ [44], the single, chromatin‐remodeling/p300‐dependent GRE of MKP1 [45], and the tethering‐based, GRE‐independent mechanism of ANXA1 [46]. Each of these architectures confers differential sensitivity to cAMP/CREB‐driven coactivator (CBP/p300) competition at the GR complex [47].

Beyond this heterogeneity in the promoter architecture, the gene‐specific differences we observed in U937‐H_2_R cells may also reflect differential coregulator recruitment and differences in the relative stoichiometry of GPCRs, G‐proteins, and GR, variables that we have previously defined as determinants of ligand‐ and context‐dependent GR transcriptional outcomes [42]. Under this framework, the higher H_2_R expression in U937‐H_2_R cells would be expected to shift the local balance of Gβγ subunits, coactivator availability (including CBP/p300), and GR occupancy in a gene‐specific manner, providing a plausible mechanistic basis for the differential regulation of GILZ, MKP1, and ANXA1 reported here.

These findings may be relevant to AML treatment. Standard AML management consists of induction chemotherapy (typically a combination of cytarabine and anthracycline) followed by post‐remission therapy, with central nervous system prophylaxis used less frequently to prevent relapses. Although approximately 70% of patients achieve remission with induction therapy, nearly all will eventually relapse without adequate post‐remission treatment. Notably, the addition of dexamethasone to intensive chemotherapy has been shown to reduce relapse and improve overall survival in hyperleukocytic AML, and glucocorticoid‐based combinations have achieved complete remissions in patients refractory to conventional agents [10, 48, 49].

We found that low‐dose dexamethasone increased U937 cells' proliferation, while higher doses reduced it. In vitro studies have reported that pediatric AML cells were prone to glucocorticoid‐induced proliferation, mainly in AML‐M5 patients and those carrying the FLT3 mutation, the most frequent mutation observed in AML [50, 51]. Cell proliferation induced by low doses of dexamethasone glucocorticoids observed herein seems to be related to increased PI3K‐Akt–mTOR pathway activity and is dampened by amthamine, which reduces its activation. This pathway has been described as critical for leukemic cell proliferation [52, 53]. Indeed, rapamycin, when combined with chemotherapy, reduced cell proliferation and induced apoptosis [54, 55].

This may be mechanistically linked to REDD1, a canonical GR target gene and mTOR repressor whose induction has been proposed to distinguish glucocorticoid‐sensitive from ‐resistant ALL cells [56]. In AML, however, constitutively elevated REDD1 is instead associated with adverse outcome [57, 58].

We therefore propose a working model in which the biphasic dexamethasone response reflects a dose‐dependent shift in REDD1 induction: at low GR occupancy, a constitutive mTOR‐activating pathway would dominate without sufficient REDD1 transactivation, yielding proliferation; at higher occupancy, REDD1 induction would override this pathway, shifting the response toward inhibition—consistent with the biphasic curves observed here. Within this model, H_2_R may act at two points: amthamine's GR‐independent lowering of mTOR activity and its potentiation of GR activity, which could lower the occupancy threshold for REDD1 induction.

Together, these effects would be expected to abolish the low‐dose proliferative phase, as observed. We thus propose a hypothetical GR–H_2_R–PI3K/mTOR–REDD1 loop, in which histaminergic signaling converts AML's biphasic glucocorticoid response into a monotonically antiproliferative one.

AML has classically been considered glucocorticoid‐resistant, but this paradigm is being re‐examined since growing evidence indicates that, in specific contexts, dexamethasone and prednisolone can promote differentiation and chemosensitize AML cells [8, 9, 10, 11, 12]. In particular, the addition of dexamethasone to intensive chemotherapy has been associated with reduced relapse and improved survival in hyperleukocytic AML, an antileukemic activity that appears enriched in NPM1‐mutated disease, and the recent DEXAML‐02 Phase II trial has provided a first prospective clinical signal in older patients [48, 59].

Dexamethasone and cytarabine have been shown to exhibit synergistic antitumor activity in an AML xenotransplant model, surpassing the effects of either agent alone [12]. Regarding histamine, as stated in the introduction, HDC combined with interleukin‐2 (IL‐2) was approved in 2008 as maintenance therapy during first remission in AML patients [22]. Given the modulatory effect of amthamine on GR transcriptional activity, we hypothesized that its co‐administration with dexamethasone would enhance chemosensitization in AML cell lines. Contrary to this expectation, neither combined nor individual treatment increased cytarabine cytotoxicity in U937 cells, highlighting a potential limitation of this strategy in this model.

Given that glucocorticoid sensitivity reportedly rises alongside cytarabine resistance in AML models and patient samples [29], we generated cytarabine–resistant U937 cells to investigate whether dexamethasone and/or amthamine could overcome chemoresistance. We observed that while corticosteroids resensitized chemoresistant cells, the H_2_ agonist exhibits a paradoxical effect: it enhanced sensitization at low dexamethasone doses but reversed it at high doses. This suggests that combining amthamine with low corticosteroid concentrations could strategically increase the sensitivity of resistant cells. A related pattern was seen in U937 proliferation assays, where the H_2_ agonist inhibited the proliferative effects of low dexamethasone doses but had no impact at high concentrations. Once more, REDD1 expression regulation could be involved in this effect.

In conclusion, dexamethasone treatment reduced proliferation of resistant cells to levels comparable to high cytarabine concentrations, potentially allowing lower chemotherapeutic dosing. While higher dexamethasone concentrations yield stronger antiproliferative effects, they increase adverse side effects. Therefore, the sensitizing effect of amthamine at low corticosteroid doses could represent a promising strategy worth exploring for AML therapy. However, these results were obtained using a single cell line, which constitutes a limitation of our study; further investigations using additional cell lines and eventually, primary AML samples are warranted to validate and generalize these findings.

## Author Contributions

**Natalia Fernández:** investigation, writing – review and editing. **Valeria Torralba‐Agu:** investigation, formal analysis, methodology, writing – review and editing. **Federico Monczor:** conceptualization, writing – original draft, writing – review and editing, project administration, funding acquisition. **Carlos Davio:** investigation, writing – review and editing. **C. Daniel Zappia:** investigation, writing – original draft, writing – review and editing, formal analysis, data curation, supervision. **Carina Shayo:** investigation, writing – review and editing.

## Funding

This work was supported by Universidad de Buenos Aires, UBACYT‐20020190100015BA. Agencia Nacional de Promoción de la Investigación, el Desarrollo Tecnológico y la Innovación, PICT2016‐2612.

## Conflicts of Interest

The authors declare no conflicts of interest.

## Supporting information


**Figure S1:** Representative full‐length blots for different antibodies.
**Figure S2:** Effect of dexamethasone on the expression of genes related to cell proliferation and differentiation.
**Figure S3:** Full length blots corresponding to Figure 5 of the main text.

## Data Availability

The data that support the findings of this study are available from the corresponding author upon reasonable request.

## References

[1] R. H. Oakley and J. A. Cidlowski , “Cellular Processing of the Glucocorticoid Receptor Gene and Protein: New Mechanisms for Generating Tissue‐Specific Actions of Glucocorticoids,” Journal of Biological Chemistry 286 (2011): 3177–3184, 10.1074/jbc.R110.179325. 21149445 PMC3030321

[2] S. Vandevyver , L. Dejager , and C. Libert , “On the Trail of the Glucocorticoid Receptor: Into the Nucleus and Back,” Traffic 13 (2012): 364–374, 10.1111/j.1600-0854.2011.01288.x. 21951602

[3] N. C. Nicolaides , Z. Galata , T. Kino , G. P. Chrousos , and E. Charmandari , “The Human Glucocorticoid Receptor: Molecular Basis of Biologic Function,” Steroids 75 (2010): 1–12, 10.1016/j.steroids.2009.09.002. 19818358 PMC2813911

[4] L. Pagliaro , S.‐J. Chen , D. Herranz , et al., “Acute Lymphoblastic Leukaemia,” Nature Reviews. Disease Primers 10 (2024): 41, 10.1038/s41572-024-00525-x. 38871740

[5] H. Döhner , D. J. Weisdorf , and C. D. Bloomfield , “Acute Myeloid Leukemia,” New England Journal of Medicine 373 (2015): 1136–1152, 10.1056/NEJMra1406184. 26376137

[6] H. Döhner , A. H. Wei , F. R. Appelbaum , et al., “Diagnosis and Management of AML in Adults: 2022 Recommendations From an International Expert Panel on Behalf of the ELN,” Blood 140 (2022): 1345–1377, 10.1182/blood.2022016867. 35797463

[7] S. Shimony , M. Stahl , and R. M. Stone , “Acute Myeloid Leukemia: 2025 Update on Diagnosis, Risk‐Stratification, and Management,” American Journal of Hematology 100 (2025): 860–891, 10.1002/ajh.27625. 39936576 PMC11966364

[8] H. Miyoshi , M. Ohki , T. Nakagawa , and Y. Honma , “Glucocorticoids Induce Apoptosis in Acute Myeloid Leukemia Cell Lines With A t(8;21) Chromosome Translocation,” Leukemia Research 21 (1997): 45–50, 10.1016/s0145-2126(96)00089-6. 9029185

[9] N. Ozbek , E. Erdemli , G. Hiçsönmez , H. Okur , and M. Tekelioğlu , “Effects of Methylprednisolone on Human Myeloid Leukemic Cells In Vitro,” American Journal of Hematology 60 (1999): 255–259. 10203097 10.1002/(sici)1096-8652(199904)60:4<255::aid-ajh1>3.0.co;2-s

[10] G. Hiçsönmez , “The Effect of Steroid on Myeloid Leukemic Cells: The Potential of Short‐Course High‐Dose Methylprednisolone Treatment in Inducing Differentiation, Apoptosis and in Stimulating Myelopoiesis,” Leukemia Research 30 (2006): 60–68, 10.1016/j.leukres.2005.05.015. 15979702

[11] M. Farrugia , C. Cutajar , J. C. Agius , and P. S. Wismayer , “Steroids‐Has the Time Come to Extend Their Use to AML?,” Journal of the Egyptian National Cancer Institute 33 (2021): 7, 10.1186/s43046-021-00062-8. 33661420 PMC13316919

[12] H. Sun , X. Liu , L. Wang , et al., “Dexamethasone Sensitizes Acute Monocytic Leukemia Cells to Ara‐C by Upregulating FKBP51,” Frontiers in Oncology 12 (2022): 888695, 10.3389/fonc.2022.888695. 35860568 PMC9290766

[13] T. Rhen and J. A. Cidlowski , “Antiinflammatory Action of Glucocorticoids‐New Mechanisms for Old Drugs,” New England Journal of Medicine 353 (2005): 1711–1723, 10.1056/NEJMra050541. 16236742

[14] L. Kalfeist , L. Galland , F. Ledys , F. Ghiringhelli , E. Limagne , and S. Ladoire , “Impact of Glucocorticoid Use in Oncology in the Immunotherapy Era,” Cells 11 (2022): 770, 10.3390/cells11050770. 35269392 PMC8909189

[15] K. De Bosscher , S. J. Desmet , D. Clarisse , E. Estébanez‐Perpiña , and L. Brunsveld , “Nuclear Receptor Crosstalk‐Defining the Mechanisms for Therapeutic Innovation,” Nature Reviews. Endocrinology 16 (2020): 363–377, 10.1038/s41574-020-0349-5. 32303708

[16] P. Schmidt , F. Holsboer , and D. Spengler , “Beta(2)‐Adrenergic Receptors Potentiate Glucocorticoid Receptor Transactivation via G Protein Beta Gamma‐Subunits and the Phosphoinositide 3‐Kinase Pathway,” Molecular Endocrinology 15 (2001): 553–564, 10.1210/mend.15.4.0613. 11266507

[17] T. Kino , A. Tiulpakov , T. Ichijo , L. Chheng , T. Kozasa , and G. P. Chrousos , “G Protein Beta Interacts With the Glucocorticoid Receptor and Suppresses Its Transcriptional Activity in the Nucleus,” Journal of Cell Biology 169 (2005): 885–896, 10.1083/jcb.200409150. 15955845 PMC2171637

[18] T. L. Kiefer , L. Lai , L. Yuan , C. Dong , M. E. Burow , and S. M. Hill , “Differential Regulation of Estrogen Receptor Alpha, Glucocorticoid Receptor and Retinoic Acid Receptor Alpha Transcriptional Activity by Melatonin Is Mediated via Different G Proteins,” Journal of Pineal Research 38 (2005): 231–239, 10.1111/j.1600-079X.2004.00198.x. 15813899

[19] C. D. Zappia , G. Granja‐Galeano , N. Fernández , et al., “Effects of Histamine H1 Receptor Signaling on Glucocorticoid Receptor Activity. Role of Canonical and Non‐Canonical Pathways,” Scientific Reports 5 (2015): 17476, 10.1038/srep17476. 26635083 PMC4669453

[20] C. D. Zappia , V. Torralba‐Agu , E. Echeverria , C. P. Fitzsimons , N. Fernández , and F. Monczor , “Antihistamines Potentiate Dexamethasone Anti‐Inflammatory Effects. Impact on Glucocorticoid Receptor‐Mediated Expression of Inflammation‐Related Genes,” Cells 10 (2021): 3026, 10.3390/cells10113026. 34831249 PMC8617649

[21] A. Martner , F. B. Thorén , J. Aurelius , J. Söderholm , M. Brune , and K. Hellstrand , “Immunotherapy With Histamine Dihydrochloride for the Prevention of Relapse in Acute Myeloid Leukemia,” Expert Review of Hematology 3 (2010): 381–391, 10.1586/ehm.10.30. 21083028

[22] M. Brune , S. Castaigne , J. Catalano , et al., “Improved Leukemia‐Free Survival After Postconsolidation Immunotherapy With Histamine Dihydrochloride and Interleukin‐2 in Acute Myeloid Leukemia: Results of a Randomized Phase 3 Trial,” Blood 108 (2006): 88–96, 10.1182/blood-2005-10-4073. 16556892

[23] M. S. Nilsson , A. Hallner , M. Brune , et al., “Immunotherapy With HDC/IL‐2 May Be Clinically Efficacious in Acute Myeloid Leukemia of Normal Karyotype,” Human Vaccines & Immunotherapeutics 16 (2020): 109–111, 10.1080/21645515.2019.1636598. 31242079 PMC7012093

[24] F. Monczor , S. Copsel , N. Fernandez , C. Davio , and C. Shayo , “Histamine H2 Receptor in Blood Cells: A Suitable Target for the Treatment of Acute Myeloid Leukemia,” Handbook of Experimental Pharmacology 241 (2017): 141–160, 10.1007/164_2016_8. 27316911

[25] P. J. Godowski , S. Rusconi , R. Miesfeld , and K. R. Yamamoto , “Glucocorticoid Receptor Mutants That Are Constitutive Activators of Transcriptional Enhancement,” Nature 325 (1987): 365–368, 10.1038/325365a0. 3808033

[26] F. Monczor , N. Fernandez , E. Riveiro , et al., “Histamine H2 Receptor Overexpression Induces U937 Cell Differentiation Despite Triggered Mechanisms to Attenuate cAMP Signalling,” Biochemical Pharmacology 71 (2006): 1219–1228, 10.1016/j.bcp.2005.12.037. 16458858

[27] D. Hochbaum , T. Tanos , F. Ribeiro‐Neto , D. Altschuler , and O. A. Coso , “Activation of JNK by Epac Is Independent of Its Activity as a Rap Guanine Nucleotide Exchanger,” Journal of Biological Chemistry 278 (2003): 33738–33746, 10.1074/jbc.M305208200. 12783872

[28] E. Echeverría , S. Ripoll , L. Fabián , C. Shayo , F. Monczor , and N. C. Fernández , “Novel Inhibitors of Phosphorylation Independent Activity of GRK2 Modulate cAMP Signaling,” Pharmacology Research & Perspectives 10 (2022): e00913, 10.1002/prp2.913. 35184416 PMC8858223

[29] D. Malani , A. Murumägi , B. Yadav , et al., “Enhanced Sensitivity to Glucocorticoids in Cytarabine‐Resistant AML,” Leukemia 31 (2017): 1187–1195, 10.1038/leu.2016.314. 27833094 PMC5420795

[30] J. Vandesompele , K. De Preter , F. Pattyn , et al., “Accurate Normalization of Real‐Time Quantitative RT‐PCR Data by Geometric Averaging of Multiple Internal Control Genes,” Genome Biology 3 (2002): research0034.1, 10.1186/gb-2002-3-7-research0034. 12184808 PMC126239

[31] M. W. Pfaffl , A. Tichopad , C. Prgomet , and T. P. Neuvians , “Determination of Stable Housekeeping Genes, Differentially Regulated Target Genes and Sample Integrity: BestKeeper‐Excel‐Based Tool Using Pair‐Wise Correlations,” Biotechnology Letters 26 (2004): 509–515, 10.1023/b:bile.0000019559.84305.47. 15127793

[32] F. Monczor and N. Fernandez , “Current Knowledge and Perspectives on Histamine H1 and H2 Receptor Pharmacology: Functional Selectivity, Receptor Crosstalk, and Repositioning of Classic Histaminergic Ligands,” Molecular Pharmacology 90 (2016): 640–648, 10.1124/mol.116.105981. 27625037

[33] N. Alonso , A. Diaz Nebreda , F. Monczor , et al., “PI3K Pathway Is Involved in ERK Signaling Cascade Activation by Histamine H2R Agonist in HEK293T Cells,” Biochimica et Biophysica Acta 1860 (2016): 1998–2007, 10.1016/j.bbagen.2016.06.016. 27316323

[34] J. Bi , Z. Min , H. Yuan , et al., “PI3K Inhibitor Treatment Ameliorates the Glucocorticoid Insensitivity of PBMCs in Severe Asthma,” Clinical and Translational Medicine 9 (2020): 22, 10.1186/s40169-020-0262-5. 32112175 PMC7048898

[35] J. A. O. Zimmerman , M. Fang , and M. A. Pufall , “PI3Kδ Inhibition Potentiates Glucocorticoids in B‐Lymphoblastic Leukemia by Decreasing Receptor Phosphorylation and Enhancing Gene Regulation,” Cancers 16 (2024): 143, 10.3390/cancers16010143. PMC10778422 38201570

[36] N. Shimizu , N. Yoshikawa , N. Ito , et al., “Crosstalk Between Glucocorticoid Receptor and Nutritional Sensor mTOR in Skeletal Muscle,” Cell Metabolism 13 (2011): 170–182, 10.1016/j.cmet.2011.01.001. 21284984

[37] W. Kang , D. Choi , J. Roh , et al., “The Role of Cyclic Adenosine Monophosphate (cAMP) in Modulating Glucocorticoid Receptor Signaling and Its Implications on Glucocorticoid‐Related Collagen Loss,” International Journal of Molecular Sciences 24 (2023): 10180, 10.3390/ijms241210180. 37373328 PMC10299417

[38] R. A. Bakker , A. Jongejan , K. Sansuk , et al., “Constitutively Active Mutants of the Histamine H1 Receptor Suggest a Conserved Hydrophobic Asparagine‐Cage That Constrains the Activation of Class A G Protein‐Coupled Receptors,” Molecular Pharmacology 73 (2007): 94–103, 10.1124/mol.107.038547. 17959710

[39] F. Monczor , N. Fernandez , B. L. Legnazzi , et al., “Tiotidine, a Histamine H2 Receptor Inverse Agonist That Binds With High Affinity to an Inactive G‐Protein‐Coupled Form of the Receptor. Experimental Support for the Cubic Ternary Complex Model,” Molecular Pharmacology 64 (2003): 512–520, 10.1124/mol.64.2.512. 12869657

[40] N. Alonso , F. Monczor , E. Echeverría , C. Davio , C. Shayo , and N. Fernández , “Signal Transduction Mechanism of Biased Ligands at Histamine H _2_ Receptors,” Biochemical Journal 459 (2014): 117–126, 10.1042/BJ20131226. 24417223

[41] N. Alonso , C. D. Zappia , M. Cabrera , et al., “Physiological Implications of Biased Signaling at Histamine H2 Receptors,” Frontiers in Pharmacology 6 (2015): 45, 10.3389/fphar.2015.00045. 25805997 PMC4354273

[42] F. Monczor , A. Chatzopoulou , C. D. Zappia , R. Houtman , O. C. Meijer , and C. P. Fitzsimons , “A Model of Glucocorticoid Receptor Interaction With Coregulators Predicts Transcriptional Regulation of Target Genes,” Frontiers in Pharmacology 10 (2019): 214, 10.3389/fphar.2019.00214. 30930776 PMC6425864

[43] L. A. Smets , G. Salomons , and J. van den Berg , “Glucocorticoid Induced Apoptosis in Leukemia,” Advances in Experimental Medicine and Biology 457 (1999): 607–614, 10.1007/978-1-4615-4811-9_67. 10500840

[44] K. A. Muzikar , N. G. Nickols , and P. B. Dervan , “Repression of DNA‐Binding Dependent Glucocorticoid Receptor‐Mediated Gene Expression,” Proceedings of the National Academy of Sciences of the United States of America 106 (2009): 16598–16603, 10.1073/pnas.0909192106. 19805343 PMC2744631

[45] L. E. Shipp , J. V. Lee , C.‐Y. Yu , et al., “Transcriptional Regulation of Human Dual Specificity Protein Phosphatase 1 (DUSP1) Gene by Glucocorticoids,” PLoS One 5 (2010): e13754, 10.1371/journal.pone.0013754. 21060794 PMC2966426

[46] S. R. Donnelly and S. E. Moss , “Functional Analysis of the Human Annexin I and VI Gene Promoters,” Biochemical Journal 332, no. Pt 3 (1998): 681–687, 10.1042/bj3320681. 9620870 PMC1219528

[47] T. Kino , S. K. Nordeen , and G. P. Chrousos , “Conditional Modulation of Glucocorticoid Receptor Activities by CREB‐Binding Protein (CBP) and p300,” Journal of Steroid Biochemistry and Molecular Biology 70 (1999): 15–25, 10.1016/s0960-0760(99)00100-4. 10528999

[48] S. Bertoli , M. Picard , E. Bérard , et al., “Dexamethasone in Hyperleukocytic Acute Myeloid Leukemia,” Haematologica 103 (2018): 988–998, 10.3324/haematol.2017.184267. 29519869 PMC6058767

[49] E. A. Kolb and P. G. Steinherz , “A New Multidrug Reinduction Protocol With Topotecan, Vinorelbine, Thiotepa, Dexamethasone, and Gemcitabine for Relapsed or Refractory Acute Leukemia,” Leukemia 17 (2003): 1967–1972, 10.1038/sj.leu.2403097. 14513046

[50] K. Klein , E. G. Haarman , V. de Haas , C. M. Zwaan , U. Creutzig , and G. L. Kaspers , “Glucocorticoid‐Induced Proliferation in Untreated Pediatric Acute Myeloid Leukemic Blasts,” Pediatric Blood & Cancer 63 (2016): 1457–1460, 10.1002/pbc.26011. 27093190

[51] D. Watanabe , A. Nogami , K. Okada , H. Akiyama , Y. Umezawa , and O. Miura , “FLT3‐ITD Activates RSK1 to Enhance Proliferation and Survival of AML Cells by Activating mTORC1 and eIF4B Cooperatively With PIM or PI3K and by Inhibiting Bad and BIM,” Cancers 11 (2019): 1827, 10.3390/cancers11121827. 31756944 PMC6966435

[52] W. Chen , E. Drakos , I. Grammatikakis , et al., “mTOR Signaling Is Activated by FLT3 Kinase and Promotes Survival of FLT3‐Mutated Acute Myeloid Leukemia Cells,” Molecular Cancer 9 (2010): 292, 10.1186/1476-4598-9-292. 21067588 PMC2993677

[53] I. Nepstad , K. J. Hatfield , T. H. A. Tvedt , H. Reikvam , and Ø. Bruserud , “Clonal Heterogeneity Reflected by PI3K‐AKT‐mTOR Signaling in Human Acute Myeloid Leukemia Cells and Its Association With Adverse Prognosis,” Cancers 10 (2018): 332, 10.3390/cancers10090332. 30223538 PMC6162751

[54] V. Dembitz , H. Lalic , A. Ostojic , R. Vrhovac , H. Banfic , and D. Visnjic , “The Mechanism of Synergistic Effects of Arsenic Trioxide and Rapamycin in Acute Myeloid Leukemia Cell Lines Lacking Typical t(15;17) Translocation,” International Journal of Hematology 102 (2015): 12–24, 10.1007/s12185-015-1776-2. 25758096

[55] N. Daver , R. F. Schlenk , N. H. Russell , and M. J. Levis , “Targeting FLT3 Mutations in AML: Review of Current Knowledge and Evidence,” Leukemia 33 (2019): 299–312, 10.1038/s41375-018-0357-9. 30651634 PMC6365380

[56] N. C. Wolff , R. M. McKay , and J. Brugarolas , “REDD1/DDIT4‐Independent mTORC1 Inhibition and Apoptosis by Glucocorticoids in Thymocytes,” Molecular Cancer Research 12 (2014): 867–877, 10.1158/1541-7786.MCR-13-0625. 24615339 PMC4260655

[57] F. Li , J. Miao , R. Liu , R. Zhang , and A. He , “Pan‐Cancer Analysis of DDIT4 Identifying Its Prognostic Value and Function in Acute Myeloid Leukemia,” Journal of Cancer Research and Clinical Oncology 150 (2024): 144, 10.1007/s00432-024-05676-8. 38507057 PMC10954950

[58] Z. Cheng , Y. Dai , Y. Pang , et al., “Up‐Regulation of DDIT4 Predicts Poor Prognosis in Acute Myeloid Leukaemia,” Journal of Cellular and Molecular Medicine 24 (2020): 1067–1075, 10.1111/jcmm.14831. 31755224 PMC6933361

[59] S. Bertoli , E. Bérard , P. Peterlin , et al., “Dexamethasone Added to Induction and Post‐Remission Therapy in Older Patients With Newly Diagnosed Acute Myeloid Leukemia: A Multicenter, Phase II Trial (DEXAML‐02),” Haematologica 110 (2025): 2475–2480, 10.3324/haematol.2024.286807. 39911128 PMC12485338

