# Supplementary material for: Cross‐Talk Between Histamine H2 Receptor and Glucocorticoid Receptor: Potential Implications in Acute Myeloid Leukemia Treatment
Source: Pharmacol Res Perspect. 2026 Sep 14;14(5):e70314. doi: 10.1002/prp2.70314 (PMC13575286; doi:10.1002/prp2.70314)
Supplement: Supplementary file 1 [file PRP2-14-e70314-s001.docx]

**Supplementary figures corresponding to:**

**Cross-talk between histamine H2 receptor and glucocorticoid receptor. Potential implications in acute myeloid leukemia treatment.**

Valeria Torralba-Agu ^a,^*, Natalia Fernández ^a,b^, Carina Shayo ^c^, Carlos Davio ^a,b^, C. Daniel Zappia ^a,b^, Federico Monczor ^a,b^.

^a^ Consejo Nacional de Investigaciones Científicas y Técnicas (CONICET), Instituto de Investigaciones Farmacológicas (ININFA), Universidad de Buenos Aires, Buenos Aires, 1113, Argentina.

^b^ Facultad de Farmacia y Bioquímica, Universidad de Buenos Aires, Buenos Aires, 1113, Argentina.

^c^ Laboratorio de Patología y Farmacología Molecular, Instituto de Biología y Medicina Experimental, CONICET, Buenos Aires, Argentina.

Corresponding autor: Federico Monczor, Instituto de Investigaciones Farmacológicas (ININFA), Junin 956, 1113, Buenos Aires, Argentina. [monczorf@ffyb.uba.ar](mailto:monczorf@ffyb.uba.ar)

* current address: Consejo Nacional de Investigaciones Científicas y Técnicas (CONICET), Instituto de Química Biológica de la Facultad de Ciencias Exactas y Naturales (IQUIBICEN), Universidad de Buenos Aires, Buenos Aires, 1113, Argentina.

**SUPPLEMENTARY FIGURE 1. Full length blots corresponding to figure 2 of the main text (N1).**

pERK Gallein

pAKT CONTROL

pERK CONTROL

pAKT Gallein

100-

70-

55-

35-

0’	1’	3’	5’

0’	1’	3’	5’

0’	1’	3’	5’	5’

0’	1’	3’	5’

ERK Gallein

ERK CONTROL

AKT CONTROL

AKT Gallein

0’	1’	3’	5’

0’	1’	3’	5’

0’	1’	3’	5’	5’

0’	1’	3’	5’	5’

100-

70-

55-

35-

pS6K Gallein

pS6K CONTROL

100-

70-

55-

0’	1’	3’

0’	1’	3’

S6K CONTROL

S6K Gallein

100-

70-

55-

0’	1’	3’

0’	1’	3’

**Supplementary Figure 1.** Representative full-length blots for different antibodies.

**BIOLOGICAL REPLICATES OF THE EXPERIMENTS CORRESPONDING TO FIGURE 2 (N2)**

pERK Gallein

pAKT Gallein

pERK CONTROL

pAKT CONTROL

100-

70-

55-

35-

0’	1’	3’	5’

0’	1’	3’	5’

0’	1’	3’	5’

0’	1’	3’	5’	5’

AKT CONTROL

100-

70-

55-

35-

AKT Gallein

ERK CONTROL

ERK Gallein

0’	1’	3’	5’

0’	1’	3’	5’

0’	1’	3’	5’	5’

0’	1’	3’	5’	5’

pS6K CONTROL

100-

70-

55-

pS6K Gallein

0’	1’	3’

0’	1’	3’

S6K CONTROL

S6K Gallein

100-

70-

55-

0’	1’	3’

0’	1’	3’

**BIOLOGICAL REPLICATES OF THE EXPERIMENTS CORRESPONDING TO FIGURE 2 (N3)**

pERK Gallein

pAKT Gallein

pERK CONTROL

pAKT CONTROL

100-

70-

55-

35-

0’	1’	3’	5’

0’	1’	3’	5’

0’	1’	3’	5’

0’	1’	3’	5’	5’

AKT CONTROL

100-

70-

55-

35-

AKT Gallein

0’	1’	3’	5’	5’

0’	1’	3’	5’	5’

pS6K CONTROL

100-

70-

55-

pS6K Gallein

0’	1’	3’

0’	1’	3’

S6K CONTROL

S6K Gallein

100-

70-

55-

0’	1’	3’

0’	1’	3’

**SUPPLEMENTARY FIGURE 2. Effect of dexamethasone on the expression of genes related to cell proliferation and differentiation.**

**Supplementary figure 2.** Analysis of GADD45β, CDKN1A and CD14 gene expression in U937 cells treated with the indicated concentrations of dexamethasone for 6 h. mRNA levels were quantified as described in Materials and Methods. Results represent the mean ± SEM of three independent experiments performed in triplicate. *p<0.05; **p<0.01; ***p<0.001.

**SUPPLEMENTARY FIGURE 3. Full length blots corresponding to figure 5 of the main text.**

pS6K

tS6K
